# Validation of an Arabic Version of the Self-Efficacy for Appropriate Medication Use Scale

**DOI:** 10.3390/ijerph182211983

**Published:** 2021-11-15

**Authors:** Hawazin Alhazzani, Ghaida AlAmmari, Nouf AlRajhi, Ibrahim Sales, Amr Jamal, Turky H. Almigbal, Mohammed A. Batais, Yousif A. Asiri, Yazed AlRuthia

**Affiliations:** 1 Department of Clinical Pharmacy, College of Pharmacy, King Saud University, P.O. Box 2454, Riyadh 11451, Saudi Arabia; Hawazin.sah@gmail.com (H.A.); ghaida.alammari@outlook.com (G.A.); 436204108@student.ksu.edu.sa (N.A.); isales@ksu.edu.sa (I.S.); yasiri@KSU.EDU.SA (Y.A.A.); 2 Family and Community Medicine Department, College of Medicine, King Saud University, P.O. Box 3145, Riyadh 12372, Saudi Arabia; amrjamal@KSU.EDU.SA (A.J.); almogbal@yahoo.com (T.H.A.); drmohammed34@gmail.com (M.A.B.); 3 Pharmacoeconomics Research Unit, Department of Clinical Pharmacy, College of Pharmacy, King Saud University, P.O. Box 2454, Riyadh 11451, Saudi Arabia

**Keywords:** medication adherence, self-efficacy, health literacy, validation studies, surveys and questionnaires, 13-item SEAMS

## Abstract

Background: Medication adherence is essential for optimal treatment outcomes in patients with chronic diseases. Medication nonadherence compromises patient clinical outcomes and patient safety as well as leading to an increase in unnecessary direct and indirect medical costs. Therefore, early identification of non-adherence by healthcare professionals using medication adherence scales should help in preventing poor clinical outcomes among patients with chronic health conditions, such as diabetes and hypertension. Unfortunately, there are very few validated medication adherence assessment scales in Arabic. Thus, the aim of this study was to validate a newly translated Arabic version of the Self-Efficacy for Appropriate Medication Use Scale (SEAMS) among patients with chronic diseases. Methods: In this single-center cross-sectional study that was conducted between March 2019 and March 2021 at the primary care clinics of King Saud University Medical City (KSUMC) in Riyadh, Saudi Arabia, the English version of SEAMS was translated to Arabic using the forward–backward method and piloted among 22 adults (≥18 yrs.) with chronic diseases. The reliability of the newly translated scale was examined using the test–retest and Cronbach’s alpha methods. Exploratory and confirmatory factor analyses were conducted to examine the construct validity of the Arabic version of SEAMS. Results: The number of patients who consented to participate and filled out the questionnaire was 202. Most of the participants were males (69.9%), aged ≥50 years (65.2%), and had diabetes (96.53%). The 13-item Arabic-translated SEAMS mean score was 32.37 ± 5.31, and the scale showed acceptable internal consistency (Cronbach’s alpha = 0.886) and reliability (Intraclass correlation coefficient = 0.98). Total variance of the 13-item Arabic-SEAMS could be explained by two factors as confirmed by the factor analysis. Conclusion: The Arabic version of SEAMS should help in detecting poor self-efficacy for medication adherence among Arabic-speaking patient populations with chronic diseases, such as diabetes and hypertension. Future studies should examine its validity among more diverse patient populations in different Arabic-speaking countries.

## 1. Introduction

Shared-decision making (SDM) has become an essential component of the patient-centered care which plays a significant role in the patient receptiveness to medical information provided by healthcare providers [1]. Therefore, it influences patient adherence to prescribed treatment regimens [2]. Medication adherence has been defined by Chakrabarti et al. as “the extent to which a person’s behavior, taking medication, following a diet, and/or executing lifestyle changes, corresponds with agreed recommendations from a health care provider” [3]. Patient nonadherence to prescription medications has always been a persistent major issue affecting as many as 50% of patients with chronic health conditions and negatively influencing the outcomes of any treatment plan [4]. It can result in a significant burden to the healthcare system in terms of direct and indirect costs by increasing recurrent admissions and hospital visits due to lack of proper management and control of chronic illnesses [5]. There are numerous factors that lead to nonadherence, such as the inability to pay for medications, poor understanding of medication instructions, the complexity of the medication regimen, poor patient–provider relationship, and drug adverse effects [6].

Generally, patients suffering from chronic diseases tend to have higher rates of nonadherence to their prescribed medicines [7]. Low levels of education, illiteracy, and cultural and social beliefs about the illness and treatment have a significant effect upon medication adherence [7]. With an increasing prevalence of chronic diseases worldwide, the increasing burden of nonadherence to medication has become even more challenging [8]. In a systematic review and meta-analysis conducted by Abegaz et al. reported that 45.2% of patients with hypertension and 31.2% of hypertensive patients with comorbidities were nonadherent to their anti-hypertensive medications [9]. In another study that explored the prevalence of nonadherence among type 2 diabetes patients in Southwest Germany using a prospective cohort study data, around 23% of the surveyed patients were found to be nonadherent to their prescribed medicines [10].

Measuring medication adherence directly using biologic samples, such as blood or urine, is impractical and costly [11]. Therefore, the use of self-report scales to assess nonadherence among patients in general, and those with chronic diseases in particular, is crucial [12]. Since numerous self-reported factors have been reported by patients with chronic health conditions, such as hypertension and diabetes, to impede adherence to prescribed treatment regimens, it is important to include these factors in any self-report medication adherence assessment scales [13]. Commonly used self-report medication adherence assessment scales include the 4-item and 8-item Morisky Medication Adherence Scales (MMAS-8) [14], Medication Adherence Report Scale (MARS) [15], and the 13-item Self-Efficacy for Appropriate Medication Use Scale (SEAMS) [16]. The SEAMS is an English 13-question self-efficacy for medication adherence questionnaire that focuses on the patient’s confidence in taking their prescribed medicines as instructed by their healthcare providers. It is a three-point scale, where 3 indicates “very confident”, 2 indicates “somewhat confident”, and 1 indicates “not confident”. Scores range between 13 to 39, and the higher scores indicate higher self-efficacy for medication adherence [16]. It has been validated in other languages, such as the Portuguese; however, it has not been translated and validated into Arabic [17]. Thus, the primary objective of this study was to test the validity and reliability of a newly Arabic translated version of the 13-item SEAMS among patients with chronic diseases, such as diabetes and hypertension. Additionally, the relationship between health literacy and the 13-item Arabic SEAMS score was examined.

## 2. Methods

### 2.1. Study Design

In this cross-sectional study, the visitors of the primary care clinics at King Saud University Medical City (KSUMC)—which is a large university-affiliated tertiary care institution that mainly provides free healthcare to King Saud University employees and their relatives in Riyadh, Saudi Arabia—were approached during their regular check-up visits and invited to participate in the study. Adult patients (e.g., ≥18 years) with chronic diseases, such as diabetes, hypertension, dyslipidemia, asthma, chronic obstructive pulmonary disease, and cardiovascular disease, were included in the study. The patients’ chronic health conditions, age, and prescription medications were checked in their electronic health records. Non-Arabic speaking patients, healthy individuals without chronic health conditions, and those who reported not taking any prescription medications were excluded from the study. In order to improve the study sample representativeness, a systematic selection of the 10th patient record from the scheduled appointments for the year of 2019 until March 2020 was conducted. Then, 319 selected files were reviewed for eligibility, resulting in a final list of 264 patients who met the inclusion criteria (e.g., adults aged ≥18 years, taking prescription medications, and Arabs). Data collection was started in March 2019 and ended in March 2021 due to the COVID-19 pandemic which resulted in a postponement of many scheduled appointments. The patients in the list were invited and interviewed by three pharmacists prior or after their scheduled appointments after explaining the purpose of this research and receiving their verbal consent to participate.

### 2.2. Research Instrument Translation and Validation

SEAMS is a 13-item self-reported medication adherence scale. Each item is scored on a 3-point Likert-scale where participants are asked to choose their level of confidence in taking medications correctly under different circumstances (1 = not confident, 2 = somewhat confident, and 3 = very confident). It was designed for patients with low literacy. The total score ranges from 13 to 39 where low scores indicate a low level of confidence and high scores indicate a high level of confidence.

Prior to translation and validation, permission was obtained from the developers. The forward–backward translation method was used in which native Arabic speakers with high English proficiency levels translated SEAMS into Arabic. The 13-item Arabic translated version of SEAMS was checked for its face and content validity by all authors. Backward translation was performed by a healthcare professional whose native language is English but is proficient in Arabic. Thereafter, the preliminary version of the Arabic-SEAMS was pilot tested between 22 patients with chronic diseases to check its comprehensibility and clarity before being presented to them again after two weeks to examine its reliability using the test–retest method. The final version of 13-item Arabic SEAMS was then approved by all authors after making minor changes based on the responses of the pilot group (Appendix A).

Additionally, participants’ sociodemographic characteristics, such as age, gender, educational level, and marital status, as well as medical characteristics (e.g., chronic diseases and prescription medication number) were collected. Furthermore, health literacy was assessed using the single item literacy screener (SILS), which consisted of one question inquiring about the need of the participant for help in understanding prescription drug leaflets or patient education materials. Those who choose “sometimes”, “often”, or “always” from the five possible answers (e.g., (5) always, (4) often, (3) sometimes, (2) rarely, and (1) never) are considered having limited or marginal health literacy. On the other hand, those who choose “rarely” or “never” are considered to have adequate health literacy [18,19].

### 2.3. Statistical Analysis

The minimum sample size required for this study was estimated to be 192 subjects using GPower^®^ software version 3.1 (Heinrich Heine University, Düsseldorf, Germany) for a medium effect size (e.g., Cohen’s d = 0.3), α = 0.05, β = 0.15, a power of 85%. Therefore, a 27.27% attrition rate out of the randomly created list of 264 patients who met the inclusion criteria would be acceptable. Frequencies, percentages, and mean ± standard deviation (SD) were used to describe the participants’ characteristics and the scores of the 13-item Arabic-SEAMS. Intraclass correlation-coefficient (ICC) was used to evaluate the test–retest reliability of the Arabic-SEAMS, and the Cronbach’s alpha coefficient was used to examine its internal consistency [20,21,22]. Exploratory factor analysis was used in assessing the internal structure of the translated SEAMS. Items with a loading of 0.30 or greater were considered for inclusion in a separate factor [23]. Kaiser–Mayer–Olkin (KMO) measure of adequacy was also used, and value greater than 0.6 proves the adequacy of the sample [24]. Discriminant validity was ensured if the square root of the average variance extracted (AVE) is higher than correlation coefficient between the extracted factors [25]. Convergent validity was checked using the composite reliability with a cutoff point of ≥0.6 [26]. Statistical analysis was conducted using SAS^®^ version 9.4 (SAS^®^ institute, Cary, NC, USA).

### 2.4. Ethical Approval

This research was approved by the institutional review board of King Saud University College of Medicine (Ref. No. 19/0271/IRB, E-19-3721, 10 March 2019). No personal identifiers, such as name or medical record numbers, were collected and all respondents gave their consent to participate. Only the authors of the study had access to the data, and the study adhered to the ethical principles of the Helsinki declaration [27].

## 3. Results

The number of patients who were recruited to this study was 202, resulting in a 76.52% response rate. Most of the patients were males (69.9%), Saudi nationals (98%), living in Riyadh (80.7%), aged between 42 and 65 years (56.9%), married (75.2%), had a post-secondary diploma or higher (50.9%), and had adequate health literacy (79.2%). The most common chronic diseases among participants were diabetes (96.53%), dyslipidemia (48.01%), and hypertension (49.01), and 57.9% of the study population were taking six medications or more (Table 1).

Item analysis of the 13-item Arabic translated SEAMS scale is presented in Table 2. The intraclass correlation coefficient (ICC) of the translated scale was 0.975 indicating excellent reliability. The mean item scores ranged between 2.24 and 2.74, while the mean total score was 32.36 with a standard deviation of 5.31. Convergent validity was ensured by having a composite reliability of 0.95, which is higher than the cutoff point of ≥0.6, and the square root of the AVEs for the two extracted factors were 0.71 and 0.65 for factor-1 and factor-2, respectively, which ensures discriminant validity since the AVEs for the two factors are higher than the correlation coefficient between the two factors (e.g., 0.59). Older age (r = 0.201, *p* = 0.004), dyslipidemia (r = 0.165, *p* = 0.018), and adequate health literacy (r = 0.238, *p* = 0.0006) were positively associated with higher SEAMS scores.

The item-total correlation coefficient has shown moderate to strong correlation of all 13 items to the total scale, ranging from 0.48 to 0.82. The Cronbach’s alpha of the overall scale was 0.88, indicating good internal consistency, and the deletion of any item on the scale did not result in improved internal consistency. Using the principal component analysis with varimax rotation, two factors were extracted from the Arabic 13-item SEAMS as shown in Table 3. Factor 1 which included items 1, 2, 3, 4, 5, 6, and 7 accounted for 57.73% of the variance, while factor 2 which included items 8, 9, 10, 11, 12, and 13 accounted for 42.27% of the variance. The two factors have accounted for 100% of the total variance. The confirmatory factor analysis confirmed the goodness of fit of the two extracted factors (e.g., Bentler Comparative Fit Index = 0.98, and RMSEA < 0.001).

## 4. Discussion

The 13-item SEAMS is an effective and reliable self-report scale to measure self-efficacy for medication adherence that is useful in assessing poor or low self-efficacy for medication adherence among patients with chronic health conditions with limited health literacy [16]. It has been translated and validated into different languages, such as Portuguese [17], Taiwanese [28], Thai [29], and Chinese [30]. However, it does not exist in Arabic, which is the world’s sixth most spoken language [31]. Sadly, Arabic speaking patients do not get their self-efficacy for medication adherence to prescribed medicines checked regularly due to the absence of valid and freely available scales [32]. In this study, the 13-item Arabic translated SEAMS demonstrated good validity and reliability. This was shown in the factor analysis that extracted two factors. The first factor included items 1, 2, 3, 4, 5, 6, and 7, while the second factor included items 8, 9, 10, 11, 12, and 13. These factors were represented in the original paper as two clear dimensions, self-efficacy for taking medications under difficult circumstances (factor 1) and self-efficacy for continuing to take medications when circumstances surrounding medication-taking are uncertain (factor 2) [16]. Additionally, it had good internal consistency with a Cronbach’s alpha of 0.88 [33], which is quite similar to the one reported for the original English scale (Cronbach’s alpha = 0.89) [16]. Moreover, the Arabic SEAMS reliability was further confirmed in the test–retest with an ICC of 0.97. This shows that the Arabic SEAMS has comparable reliability, if not higher, to other medication adherence and self-efficacy for medication adherence assessment scales that have been validated into Arabic, but are not easily accessible or freely available, such as the MMAS-8 and MARS [32,33,34].

Health literacy was positively associated with higher SEAMS scores, which means that patients with adequate health literacy were more likely to adhere to their prescribed medicines than those with limited health literacy. This is consistent with the preponderance of evidence that shows a positive correlation between health literacy and medication adherence [35]. Interestingly, dyslipidemia was positively associated with higher SEAMS score. This finding may be related to the fact that most patients with dyslipidemia in the study were on statins, such as atorvastatin and simvastatin. The use of statins was positively associated with a higher medication adherence among a sample of Arabic-speaking patients with dyslipidemia in Jordan [36]. Another interesting finding was the positive association between older age and SEAMS score. Although many tend to think that adherence declines with age [37], other studies suggest that older adults (e.g., ≥65 yrs.) are more likely to adhere to their prescribed treatment regimens in comparison to their younger counterparts [38].

Although this is the first study to the best of our knowledge that translated and validated the 13-item SEAMS into Arabic, some limitations should be acknowledged. First, the majority of patients in the study had adequate health literacy, contrary to the original study that evaluated the psychometric properties of the scale among low-literacy patients [16]. Therefore, the comprehensibility of the translated scale needs to be assessed further among patients with low-literacy levels. Moreover, health literacy was assessed using the single item literacy screener (SILS). Though SILS is a valid tool to assess health literacy, it is not as reliable as other scales [19], such as the 67-item Test of Functional Health Literacy (TOFHL), which was not used due to the time constraint associated with administering such a lengthy questionnaire [39]. Additionally, this was a single center study and the majority of patients were Saudi (98%) which may limit the generalizability of the study findings. However, the Arabic language and terms used can be easily understood by any native Arabic speaker. Additionally, the Arabic-SEAMS was not cross-checked using other adherence assessment methods, such as pill counts and medication possession ratios [11].

## 5. Conclusions

The 13-item Arabic-translated SEAMS showed acceptable levels of validity and reliability, which makes it a valid option to assess self-efficacy for medication adherence among Arabic-speaking patients with different chronic health conditions and variable levels of health literacy. Furthermore, it should aid in improving patient care for native Arabic-speaking patients and minimize unnecessary costs and overall burden. Future studies should examine the psychometric properties of the Arabic-SEAMS among larger samples of patients with chronic health conditions, and particularly those with low-literacy levels. Additionally, the concurrent validity of the Arabic-SEAMS should be examined using other valid adherence measures, such as pill counts or medication possession ratios.

## Figures and Tables

**Table 1:** Study participants’ sociodemographic characteristics (*n* = 202).

Gender	
Male	141 (69.80)
Female	61 (30.20)
Age group (years)	
18–25	22 (10.89)
26–33	11 (5.45)
34–41	14 (6.93)
42–49	23 (11.39)
50–57	34 (16.83)
58–65	58 (28.71)
>65	40 (19.80)
Marital status	
Single	30 (14.85)
Married	152 (75.25)
Divorced/widowed	20 (9.90)
Educational level	
No official education	32 (15.84)
Elementary school diploma	13 (6.44)
Middle school diploma	15 (7.43)
Secondary school diploma	39 (19.31)
Associate degree	15 (7.43)
Bachelor of Science or equivalent	76 (37.62)
Graduate degree (master of science or doctor of philosophy)	12 (5.94)
Chronic health conditions	
Diabetes	195 (96.53)
Hypertension	99 (49.01)
Dyslipidemia	97 (48.01)
Asthma	15 (7.43)
Cardiovascular disease	12 (5.94)
Number of prescription medications	
1–2	32 (15.84)
3–4	35 (17.33)
5–6	18 (8.91)
>6	117 (57.92)
Health literacy	
Adequate	160 (79.21)
Marginal/limited	42 (20.79)

**Table 2:** Item analysis of the 13-item Arabic-SEAMS.

Item	Mean ± SD	Item–Total Correlation	Cronbach’s α If Item Deleted
1- When you take several different medicines each day?	2.74 ± 0.52	0.56	0.878
2- When you have a busy day planned?	2.57 ± 0.56	0.62	0.875
3- When you are away from home?	2.51 ± 0.64	0.67	0.873
4- When no one reminds you to take the medicine?	2.57 ± 0.64	0.57	0.877
5- When you take medicines more than once a day?	2.63 ± 0.58	0.66	0.873
6- When the schedule to take the medicine is not convenient?	2.49 ± 0.64	0.57	0.878
7- When your normal routine gets messed up?	2.47 ± 0.60	0.68	0.872
8- When you get a refill of your old medicines and some of the pills look different than usual?	2.38 ± 0.70	0.49	0.881
9- When you are not sure how to take the medicine?	2.24 ± 0.71	0.49	0.882
10- When you are not sure what time of the day to take your medicine?	2.33 ± 0.65	0.55	0.879
11- When a doctor changes your medicines?	2.57 ± 0.61	0.50	0.881
12- When they cause some side effects?	2.19 ± 0.66	0.48	0.883
13- When you are feeling sick (like having a cold or the flu)?	2.60 ± 0.59	0.56	0.879

**Table 3:** Factor Analysis of the 13-item Arabic-SEAMS (Varimax rotation method).

Item	Factor-1	Factor-2
1- When you take several different medicines each day?	**0.708**	
2-When you have a busy day planned?	**0.815**	
3- When you are away from home?	**0.750**	
4- When no one reminds you to take the medicine?	**0.643**	
5- When you take medicines more than once a day?	**0.780**	
6- When the schedule to take the medicine is not convenient?	**0.621**	
7- When your normal routine gets messed up?	**0.677**	
8- When you get a refill of your old medicines and some of the pills look different than usual?		**0.532**
9- When you are not sure how to take the medicine?		**0.814**
10- When you are not sure what time of the day to take your medicine?		**0.798**
11- When a doctor changes your medicines?		**0.478**
12- When they cause some side effects?		**0.668**
13- When you are feeling sick (like having a cold or the flu)?		**0.537**

## Data Availability

The data are available upon reasonable request from the corresponding. Author (Yazed AlRuthia).

## Appendix A

**Table A1:** Self-Efficacy for Appropriate Medication Use Scale (SEAMS).

How Confident Are You That You Can Take Your Medicines Correctly?	Not at All Confident	Somewhat Confident	Very Confident
1. When you take several different medicines each day?	☐	☐	☐
2. When you have a busy day planned?	☐	☐	☐
3. When you are away from home?	☐	☐	☐
4. When no one reminds you to take the medicine?	☐	☐	☐
5. When you take medicines more than once a day?	☐	☐	☐
6. When the schedule to take the medicine is not convenient?	☐	☐	☐
7. When your normal routine gets messed up?	☐	☐	☐
8. When you get a refill of your old medicines and some of the pills look different than usual?	☐	☐	☐
9. When you are not sure how to take the medicine?	☐	☐	☐
10. When you are not sure what time of the day to take your medicine?	☐	☐	☐
11. When a doctor changes your medicines?	☐	☐	☐
12. When they cause some side effects?	☐	☐	☐
13. When you are feeling sick (like having a cold or the flu)?	☐	☐	☐
